# Clinicopathologic Features and Prognostic Implications of Golgi Phosphoprotein 3 in Non-small Cell Lung Cancer: A Meta-analysis

**DOI:** 10.7150/jca.30067

**Published:** 2019-10-04

**Authors:** Wenhua Shi, Wei Feng, Jian Wang, Cui Zhai, Qianqian Zhang, Qingting Wang, Yang Song, Xin Yan, Limin Chai, Pengtao Liu, Yuqian Chen, Cong Li, Manxiang Li

**Affiliations:** Department of Respiratory and Critical Care Medicine, The First Affiliated Hospital of Xi'an Jiaotong University, Xi'an, Shaanxi 710061, China

**Keywords:** GOLPH3, non-small cell lung cancer, biomarker, prognosis, meta-analysis

## Abstract

**Background:** A number of studies have investigated the role of Golgi phosphoprotein-3 (GOLPH3) in the pathogenesis and progression of non-small cell lung cancer (NSCLC). However, the results of previous studies are heterogeneous and controversial. The aim of this meta-analysis was to clarify its association with the clinicopathological characteristics of patients and evaluate the prognostic significance of GOLPH3 in NSCLC.

**Methods:** A systematic search was conducted through PMC, PubMed, Medline, Web of Science, Chinese National Knowledge Infrastructure and Wanfang database. The odds ratio (OR) and hazard ratio (HR) with 95 % CI were calculated by STATA 12.0.

**Results:** 8 qualified studies with a total of 1001 patients with NSCLC were included. Pooled results showed that GOLPH3 was highly expressed in tumor tissues compared with adjacent lung tissues (OR, 7.55), and overexpression of GOLPH3 was significantly correlated with advanced clinical stage (OR, 3.42), poor differentiation of tumor (OR, 1.97) and positive lymph node metastasis (OR, 2.58), but no association with histological type, gender, age or tumor size was found in NSCLC patients. In addition, the pooled HR for overall survival was 1.79 by univariate analysis and 1.91 by multivariate analysis. The pooled HR for progression-free survival was 2.50.

**Conclusions:** GOLPH3 could be a risk factor for progression of NSCLC and might act as a potential prognostic biomarker for NSCLC patients.

## Introduction

Lung cancer is the most common malignancy and the leading cause of cancer-related death worldwide, with 234,030 new cases and 154,050 deaths estimated for the year 2018 in United States 1. Non-small-cell lung carcinoma (NSCLC) is the main pathological type of lung cancer, accounting for more than 80% of the cases 2. Clinically, the majority of NSCLC patients are often diagnosed at an advanced stage and ineligible for surgical resection with a curative intent 3, making successful treatment more difficult and overall survival poor despite the development of the molecular targeted agents 4 and the therapeutic strategy 5. It has been demonstrated that the decreased survival rate is partially determined by the subtleness of symptoms, delayed diagnosis, concomitant diseases and limited therapeutic options 6, 7. In recent years, a multitude of potentially useful biomarkers have emerged and established important applications in early diagnosis, risk stratification and prognosis assessment of lung cancer, which is helpful to implement timely intervention and improve the clinical outcome of patients.

Golgi phosphoprotein 3 (GOLPH3), a phosphorylated protein with a molecular weight of 34 kDa, is involved in anterograde and retrograde Golgi trafficking 8-10. Previous studies have demonstrated that GOLPH3 is highly expressed in various types of human cancer, including NSCLC 11, gastric cancer 12, breast cancer 13, hepatocellular cancer 14 and prostate cancer 15, and overexpression of GOLPH3 promotes cancer cell proliferation, migration and invasion^15^, ^16^. In addition, GOLPH3 gene expression has been shown to be associated with poor prognosis and chemotherapy resistance in patients with cancer 10, 17. Although quite a number of studies have investigated the clinical role of GOLPH3 in NSCLC and indicated that GOLPH3 could be a risk factor and prognostic biomarker for NSCLC, the relationships of GOLPH3 expression with clinicopathologic features in patients with NSCLC are inconsistent and controversial. Therefore, we performed a meta-analysis including all eligible studies to reveal the authentic value of GOLPH3 in cancer outcome and clinicopathological characteristics thereby providing more evidence for clinical practice.

## Methods

### Search strategy

Articles were identified using a comprehensive literature search in PMC, Pubmed, Medline, Web of Science, Chinese National Knowledge Infrastructure (CNKI) and Wanfang databases. The time scope that we defined was from databases inception to January 2019. The combination of the Medical Subject Headings (MeSH) and the keywords that we used for searching literature was: (“lung cancer” or “lung tumor” or “lung carcinoma” or “NSCLC” or “non-small cell lung cancer”) and (“GPP34 protein” or “Golgi phosphoprotein 3” or “GOLPH3”). The relevant reviews and the references of the included studies were also examined manually to find additional data.

### Inclusion and exclusion criteria of literature

Studies included in this meta-analysis were required to meet the following criteria: (1) cancers were diagnosed histologically; (2) the expression of GOLPH3 was evaluated by immunohistochemistry; (3) clinical and histological data of NSCLC patients were provided; (4) carcinoma specimens were taken prior to chemotherapy, radiotherapy and other drug treatment; (5) articles had to be published in English or Chinese. The exclusion criteria: (1) studies that contained duplicated data; (2) editorials, letters, conference proceedings, comments and case reports; (3) studies without relevant data; (4) studies not performed in humans.

### Data extraction and quality assessment

Two independent reviewers extracted all the available data from each eligible study. The following information were extracted: author, publication year, GOLPH3 assessment method, follow-up period, numbers of patients, case number of different groups, age of patients, tumor size, cut-off values for age and tumor size, histological classification, tumor differentiation degree, tumor node metastases, T descriptor, tumor-node-metastasis (TNM) classification, hazard ratio (HR) and corresponding 95% confidence intervals (CI) for OS and PFS. The HRs (95 % CIs) by univariate analysis and multivariate analysis were extracted directly from some studies. When HR and 95% CI were not provided in the article, these values were estimated from the Kaplan-Meier curves using the method described previously 18, 19. Quality assessment of included studies was performed by two independent authors based on Newcastle-Ottawa Scale grading system 20. Any disagreements were solved by discussion with a third team member.

### Statistical analysis

We calculated the OR and 95% CI to determine the association of GOLPH3 expression and clinicopathological characteristics in patients with NSCLC. If OR > 1 and the 95% CI doesn't include 1, it indicates that there is an association between high GOLPH3 expression and clinicopathological variable. The impact of GOLPH3 overexpression on OS and PFS of NSCLC patients was estimated by HR and its 95% CI and HR > 1 represents a poor prognosis. We performed this meta-analysis using two different approaches (fixed and random effects model analysis) based on heterogeneity. Potential heterogeneity among the studies was quantified using chi-squared test and I^2^ 21, which was defined statistical significance at a level of p < 0.1 or I^2^ > 50%. A fixed effects model was selected in the absence of significant heterogeneity. Otherwise, a random effects model was applied 22. If necessary, we performed subgroup analysis to explore potential sources of heterogeneity. Egger's test was implemented to visually identify publication bias 23, and sensitivity analysis was conducted by eliminating each study to examine the robustness of the results. All statistical analyses were performed using STATA software (Version 12.0; STATA Corporation, College Station, TX), and a two-sided p value less than 0.05 was considered statistically significant.

## Results

### Searching of literature

**Figure 1:**
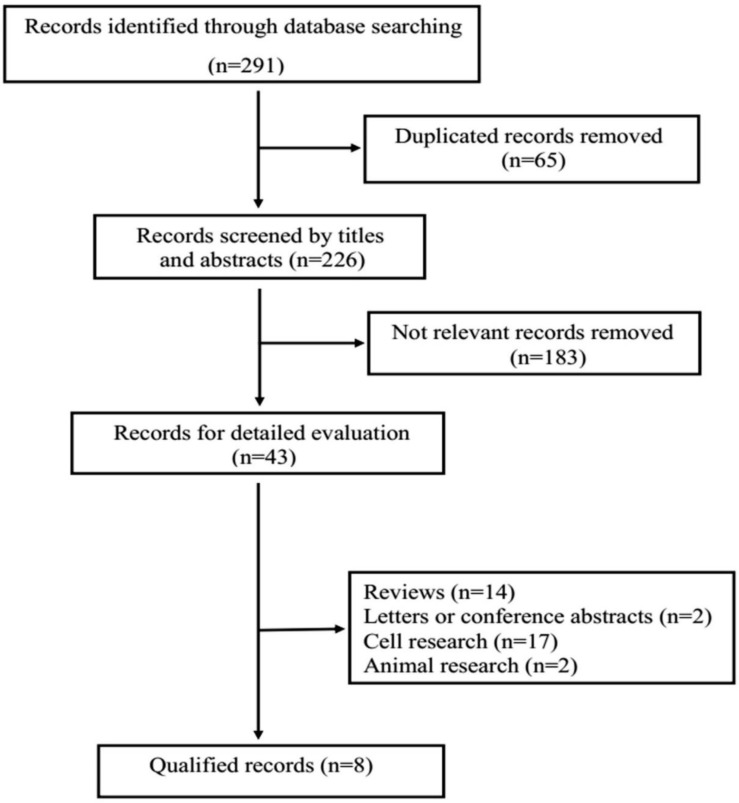
Flow chart of the selection for the meta-analysis.

The diagram of the study selection process is shown in Figure 1. During the initial search of the medical literature related to GOLPH3 and lung cancer, we identified 291 studies. Among these articles, 65 duplicate studies and 183 irrelevant studies were excluded after reviewing title and abstracts of articles. Subsequently, 43 articles were excluded for the following reasons: reviews (n=14), letters or conference abstracts (n=2), cell research (n=17), animal research (n=2). Finally, 8 articles met the inclusion criteria and were included for further analysis. A manual search of the reference lists of the 8 studies did not yield any eligible studies.

### Characteristics of included studies

As shown in Table 1, a total of eight studies 11, 16, 24-29 with 1001 newly diagnosed NSCLC patients were assessed for this analysis. All eligible studies were published from 2014 to 2018 in Asia, with a sample size ranging from 57 to 230, and the median follow-up of the studies was 33.9-52 months. All the studies were retrospective study design and 33.9-74.0% of the patients were male. The expression of GOLPH3 in cancer and adjacent normal tissues from NSCLC patients was detected by immunohistochemistry (IHC) staining, which included assignment of staining intensity and area of positive staining using the semi-quantitative scoring system. The histological classification of lung cancer mainly contained lung adenocarcinoma (518 patients) and lung squamous cell carcinoma (458 patients). The quality of included studies assessed by Newcastle-Ottawa scale was considered acceptable, as all the studies had more than 5 stars of scores (Table 1).

### GOLPH3 expression in cancer and adjacent normal lung tissues

**Figure 2:**
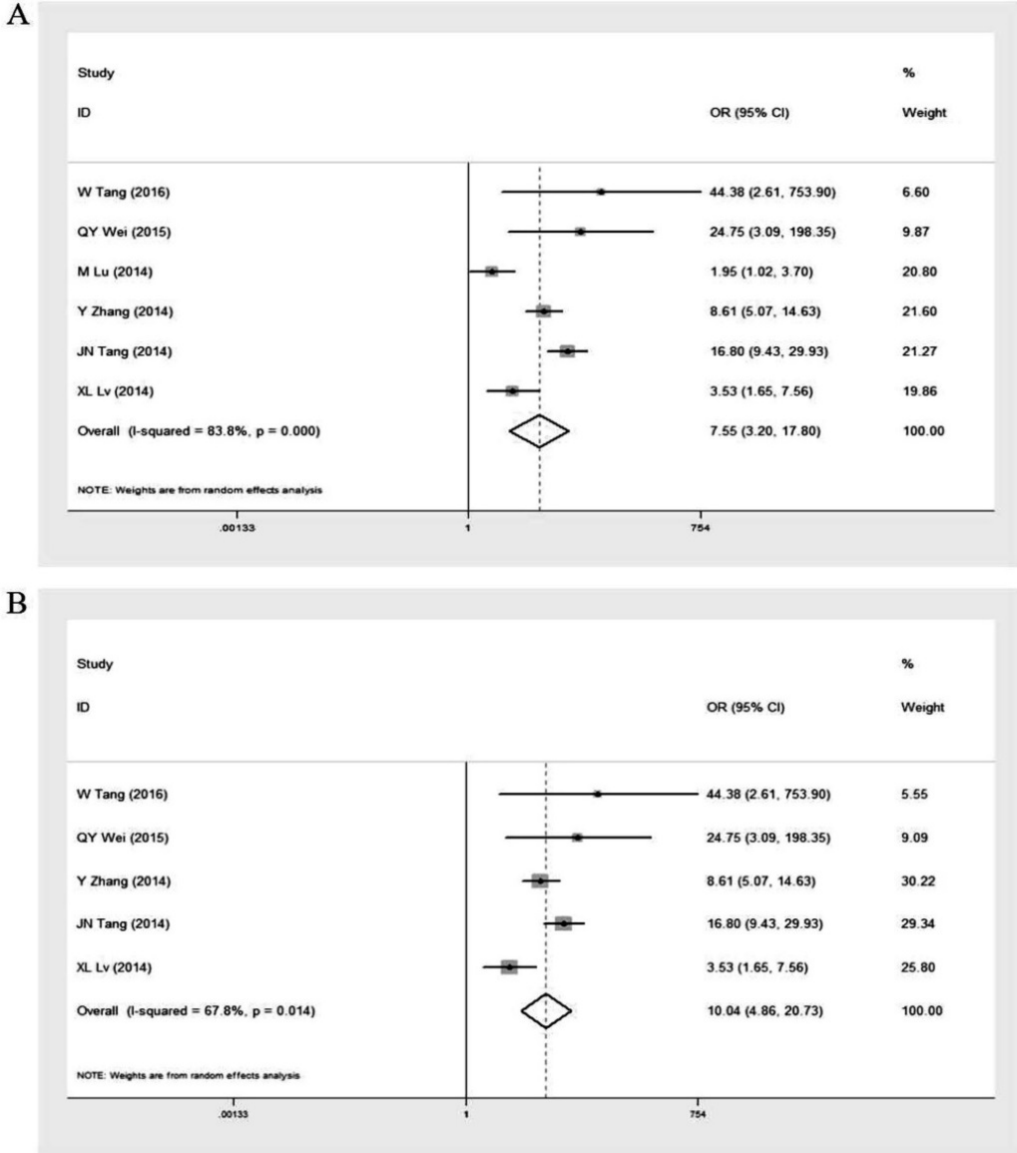
Forest plot of odds ratio (OR) for GOLPH3 expression in lung cancer tissue samples and adjacent normal lung tissue samples. **A.** including the study by M Lu et al.; **B.** excluding the study by M Lu et al. cancer tissue vs adjacent normal tissue. If OR > 1 and the 95% CI doesn't include 1, it indicates that expression level of GOLPH3 was dramatically higher in the cancer tissues of NSCLC patients compared with adjacent lung tissues.

Six studies 16, 25-29 provided GOLPH3 expression data in 711 lung cancer tissue samples and 461 adjacent normal lung tissue samples (Table 2). The overall OR was 7.55 (95% CI = 3.20-17.80, p < 0.001) via a random effects model analysis (I^2^ = 83.8%, p < 0.001) (Figure 2A). After excluding the study by Ming Lu et al. 26 which was performed in patients with stage I NSCLC only, the heterogeneity decreased (I^2^ = 67.8%, p = 0.014) and the overall OR was 10.04 (95% CI = 4.86-20.73, p < 0.001) (Figure 2B). These results indicated that expression level of GOLPH3 was dramatically higher in the cancer tissues of NSCLC patients compared with adjacent lung tissues and the study by Ming Lu et al. might partly contribute to the heterogeneity.

### GOLPH3 expression and clinical stage

**Figure 3:**
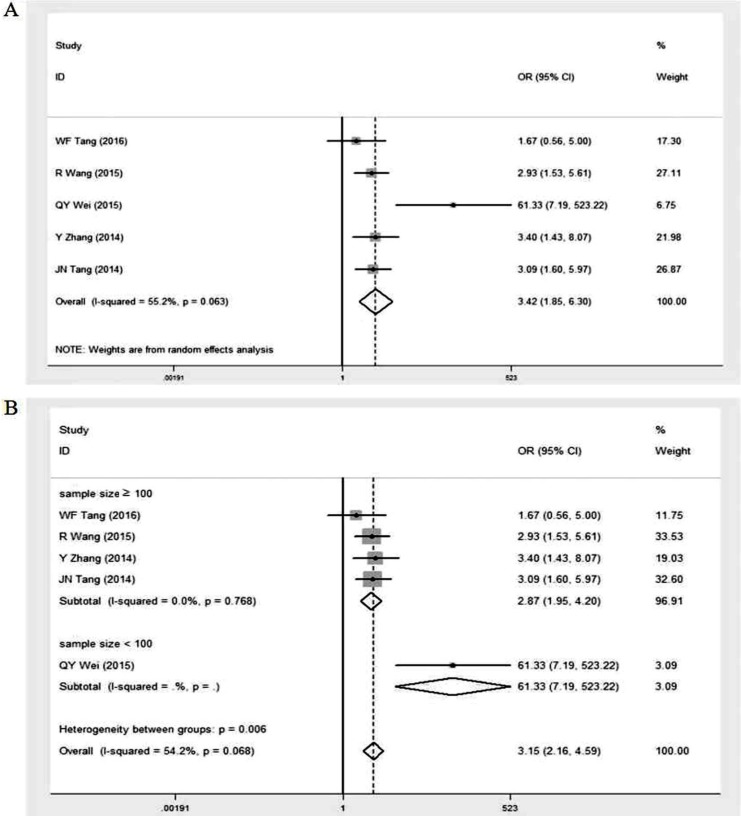
Meta-analysis of association between high GOLPH3 expression and clinical stage of lung cancer. **A.** Odds ratio (OR) with 95% CI for the association between high GOLPH3 expression and clinical stage of patients, III-IV stage vs I-II stage. **B.** Subgroup analysis of OR based on sample size, III-IV stage vs I-II stage. If OR > 1 and the 95% CI doesn't include 1, it indicates that there is an association between high GOLPH3 expression and advanced clinical stage of patients with lung cancer.

Five studies 16, 24, 25, 27, 28 reported relevant data concerning the expression of GOLPH3 in different clinical stages of NSCLC patients (Table 2). Our pooled result showed that the overall OR was 3.42 (95% CI = 1.85-6.30, p < 0.001) via a random effects model analysis (I^2^ = 55.2%, p = 0.063) (Figure 3A). Subgroup analysis stratified by sample size showed that the summary OR was 2.87 (95% CI = 1.95-4.20, p < 0.001; I^2^ = 0%, p = 0.768) in studies with sample size ≥ 100 and 61.33 (95% CI = 7.19-523.22, p < 0.001) in studies with fewer subjects (Figure 3B). Significant association existed between high GOLPH3 expression and clinical stage of patients, indicating that expression level of GOLPH3 was dramatically higher in advanced stage than that in early stage.

### GOLPH3 expression and tumor differentiation

**Figure 4:**
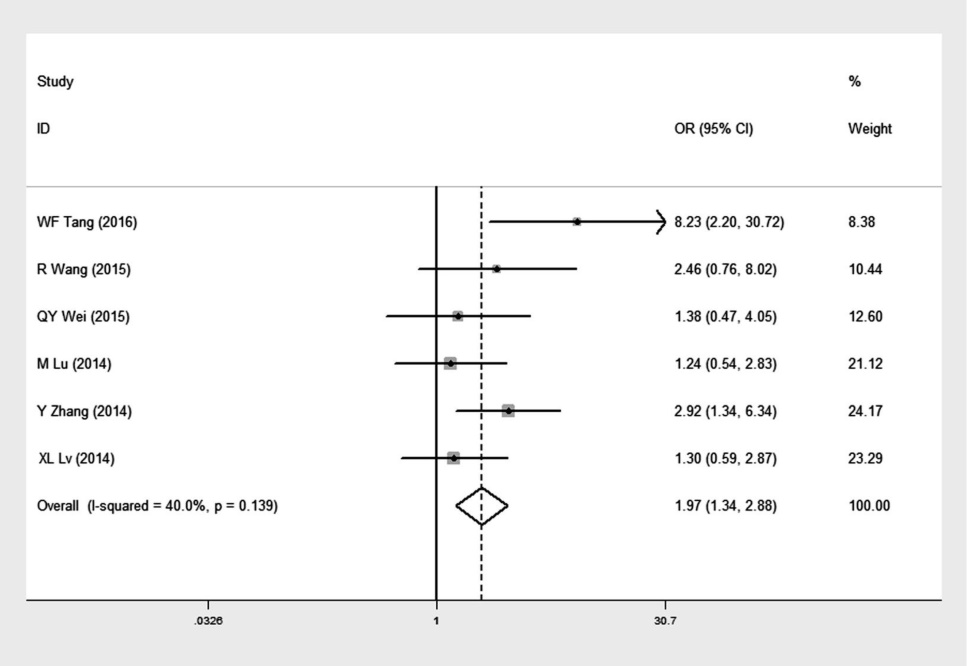
Forest plot of odds ratio (OR) with 95% CI for the association between high GOLPH3 expression and tumor differentiation in NSCLC, moderate to poor differentiation vs well differentiation. If OR > 1 and the 95% CI doesn't include 1, it indicates that there is an association between high GOLPH3 expression and poor differentiation of tumor.

Six studies 16, 24-27, 29, comprising of 765 NSCLC patients, reported data concerning the expression of GOLPH3 in different degree of tumor differentiation in NSCLC patients (Table 2). The overall OR was 1.97 (95% CI = 1.34-2.88, p= 0.002) via a fixed effects model analysis (I^2^ = 40.0%, p = 0.139) (Figure 4), suggesting that expression level of GOLPH3 was relatively higher in moderate and poor differentiation of tumor than that in well tumor differentiation.

### GOLPH3 expression and histologic type

**Figure 5:**
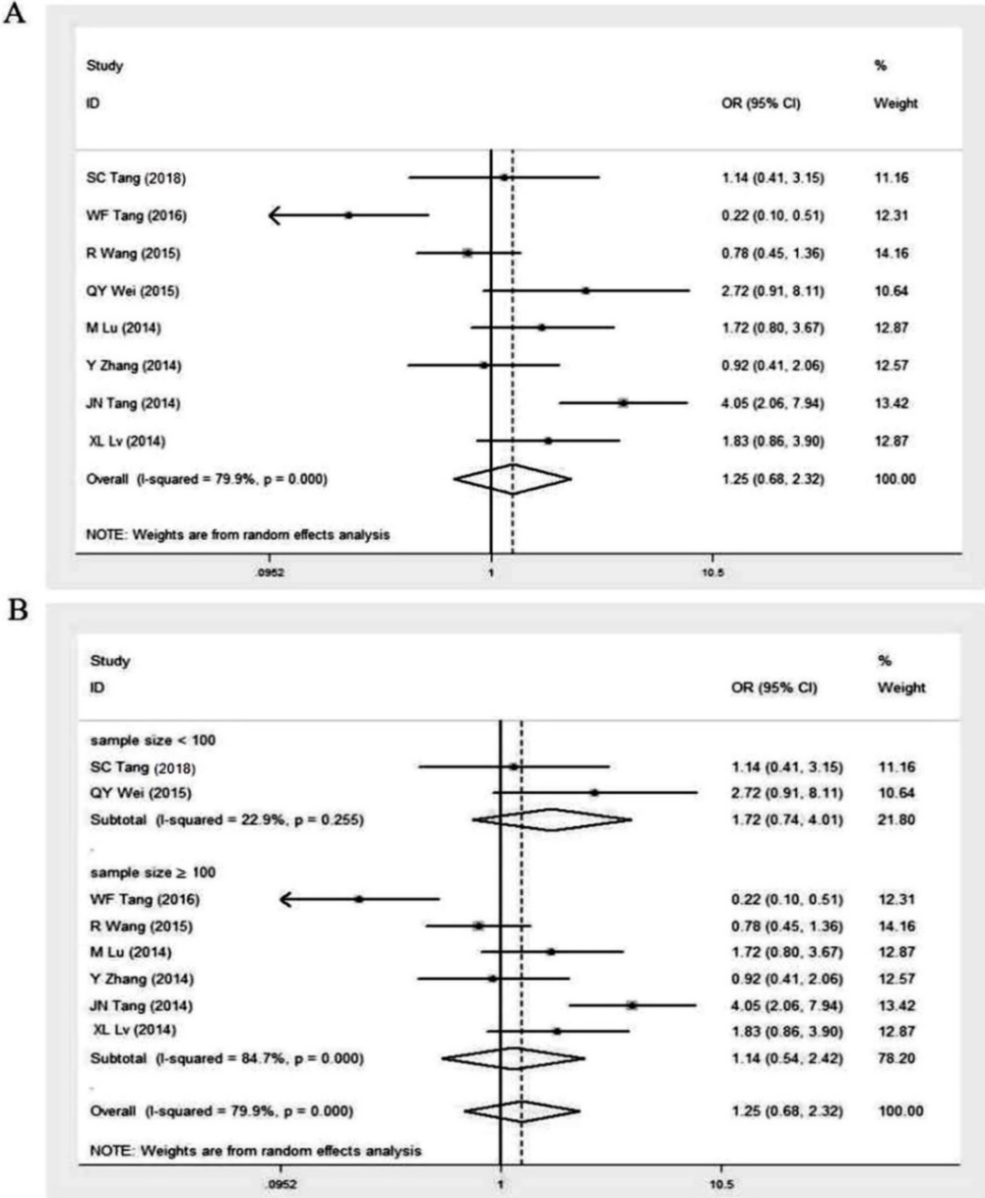
Meta-analysis of association between high GOLPH3 expression and histologic type of lung cancer. **A.** Odds ratio (OR) with 95% CI for the association between high GOLPH3 expression and histologic type of lung cancer, lung squamous cell carcinoma vs lung adenocarcinoma. **B.** Subgroup analysis of OR based on sample size, lung squamous cell carcinoma vs lung adenocarcinoma. If OR > 1 and the 95% CI doesn't include 1, it indicates that there is an association between high GOLPH3 expression and histologic type of lung cancer.

Eight studies investigated the relationship between GOLPH3 expression and histologic type of lung cancer 11, 16, 24-29. The histological classification in these studies mainly contained lung adenocarcinoma (518 patients) and lung squamous cell carcinoma (458 patients) (Table 2). The overall OR was 1.25 (95% CI = 0.68-2.32, p = 0.474) via a random effects model analysis (I^2^ = 79.9%, p < 0.001) (Figure 5A). Subgroup analysis stratified by sample size showed that the OR in studies with sample size ≥ 100 was 1.14 (95% CI = 0.54-2.42, p = 0.733; I^2^ = 84.7%, p < 0.001). And the OR in smaller studies (sample size < 100) was 1.72 (95% CI = 0.74-4.01, p = 0.211; I^2^ = 22.9%, p = 0.255) (Figure 5B). No significant association existed, indicating that GOLPH3 expression was not related to histologic type of lung cancer.

### GOLPH3 expression and lymphatic metastasis

**Figure 6:**
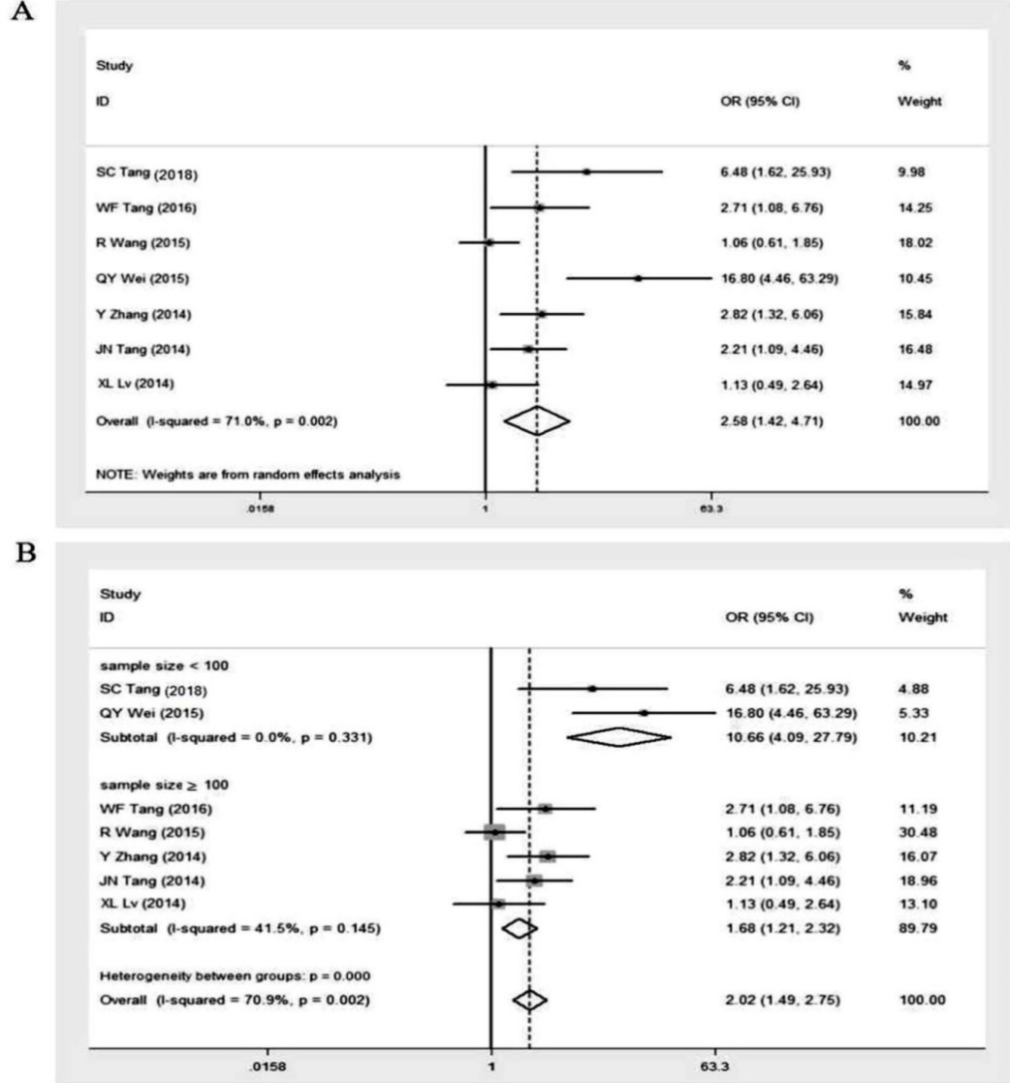
Meta-analysis of association between high GOLPH3 expression and lymphatic metastasis. **A.** Odds ratio (OR) with 95% CI for the association between high GOLPH3 expression and lymphatic metastasis, nodal metastasis vs no nodal metastasis.** B.** Subgroup analysis of OR based on sample size, nodal metastasis vs no nodal metastasis. If OR > 1 and the 95% CI doesn't include 1, it indicates that there is an association between high GOLPH3 expression and lymph node metastasis.

Seven studies 11, 16, 24, 25, 27-29 revealed the correlation between the expression level of GOLPH3 and different status of lymph node metastasis in patients with NSCLC, including 354 cases with lymphatic metastasis and 531 cases without lymphatic metastasis (Table 2). A random effects model was used due to the high heterogeneity (I^2^ = 71.0%, p = 0.002) and the overall OR was 2.58 (95% CI = 1.42-4.71, p = 0.001) (Figure 6A). Subgroup analysis stratified by sample size showed that the summary OR in studies with more than 100 subjects was 1.68 (95% CI = 1.21-2.32, p < 0.001; I^2^ = 41.5%, p = 0.145). And the OR for studies with fewer subjects (sample size < 100) was 10.66 (95% CI = 4.09-27.79, p < 0.001; I^2^ = 0.00%, p = 0.331) (Figure 6B). These results suggested that the expression level of GOLPH3 was markedly higher in lymphatic metastasis group compared to non-lymphatic metastasis group.

### Association between GOLPH3 expression and gender, age and tumor size

A total of 8 studies 11, 16, 24-29 assessed the relationship between GOLPH3 expression and general characteristics of patients (gender, age) (Table 2). The I^2^ and p-value for heterogeneity were 53.6% and 0.035, respectively. Therefore, the random effects model was used in gender analysis. Our results showed that increased GOLPH3 expression was not associated with gender of patients (OR = 1.19, 95% CI = 0.78-1.80, p = 0.42). Similar results were also found in the subgroup analyses according to sample size (n ≥ 100 or n < 100) (Table 3). Considering the use of different cut-off values for age ( 60 years in three studies 11, 16, 28 and 65 years in five studies 24-27, 29), we performed separate meta-analyses and found that the overall OR in studies using 60 years and 65 years as age cut-off was 1.13 (95% CI = 0.67-1.91, p = 0.658) and 0.96 (95% CI = 0.59-1.56, p = 0.854), respectively (Table 3) . These results indicated that there was no significant association between GOLPH3 expression and age of patients. In addition, eight studies 11, 16, 24-29 revealed the correlation between the GOLPH3 expression and tumor size. Among these studies, three studies 25, 27, 28 used 3cm as tumor size cut-off, two studies 11, 16 used a cut-off value of 5cm and others 24, 26, 29 used the T descriptor from the TNM staging system. The results showed that no significant association between high expression of GOLPH3 and tumor size was found in three subgroups. Pooled ORs and heterogeneity test results for different subgroups in tumor size analysis are available in Table 3.

### Prognosis value of GOLPH3 in malignant tumors

**Figure 7:**
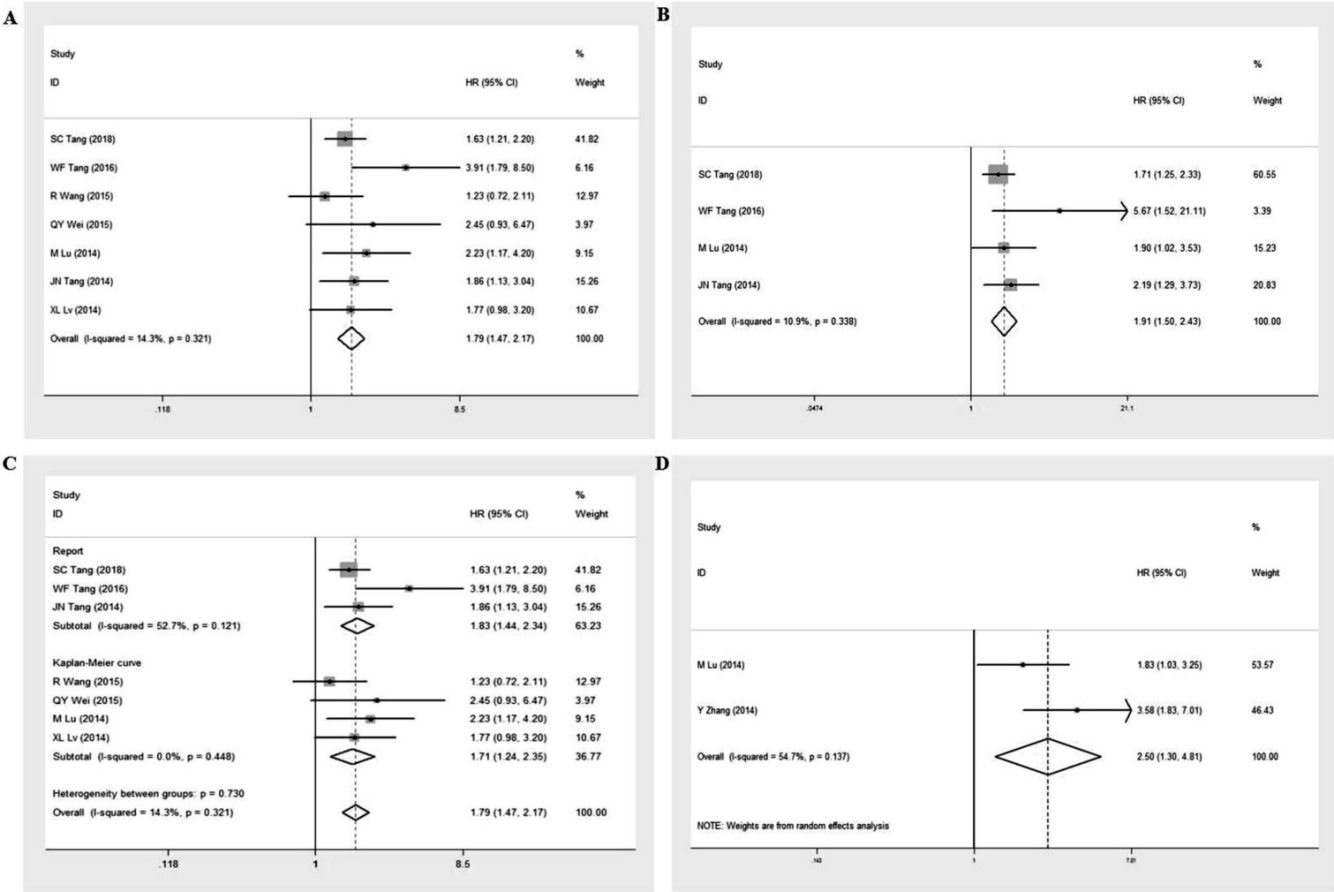
Forest plots for the prognostic meta-analysis. A. Hazard ratio (HR) with 95% CI for the association between high GOLPH3 expression and overall survival (OS) in univariate analysis, high vs low GOLPH3 expression. **B.** HR with 95% CI for the association between high GOLPH3 expression and OS in multivariate analysis, high vs low GOLPH3 expression. **C.** Subgroup analysis of HR based on the method of obtaining HRs with 95% CI for OS in univariate analysis, high vs low GOLPH3 expression. **D.** HR with 95% CI for the association between high GOLPH3 expression and progression free survival (PFS) in multivariate analysis, high vs low GOLPH3 expression. If HR > 1 and the 95% CI doesn't include 1, it represents that patients with high GOLPH3 expression have a poor prognosis.

Seven studies 11, 16, 24, 26-29 with 856 NSCLC patients were eligible to evaluate the correlation between overexpression of GOLPH3 and OS of cancer patients, including seven studies 11, 16, 24, 26-29 for univariate analysis and four studies 11, 16, 26, 28 for multivariate analysis (Table 4). The pooled HR was 1.79 (95% CI = 1.47-2.17, p < 0.001; I^2^ = 14.3%, p = 0.321) for univariate analysis and 1.91 (95% CI = 1.50-2.43, p < 0.001; I^2^ = 10.9%, p = 0.338) for multivariate analysis (Figure 7A and 7B). Considering the different method of obtaining HRs with 95%CI for OS in univariate analysis (HRs and 95% CI were extracted directly from three studies 11, 16, 28 and estimated by Kaplan-Meier survival curves in four studies 24, 26, 27, 29), we further performed a subgroup analysis based on the method of obtaining HRs. The results showed that significant relationships between high expression of GOLPH3 and poor OS were exhibited in both subgroups (HR = 1.83, 95% CI = 1.44-2.34; HR = 1.71, 95% CI = 1.24-2.35, respectively) (Figure 7C). In addition, two studies 25, 26 including 261 NSCLC patients reported the HRs with 95%CI for progression-free survival (PFS) in the multivariate models (Table 4). The pooled HR was 2.50 (95% CI = 1.30-4.81, p = 0.006) using the random effects model (I2=54.7%, p = 0.137) (Figure 7D). These results indicated that high GOLPH3 expression could predict a poor prognosis for NSCLC patients.

### Sensitivity analysis and publication bias

**Figure 8:**
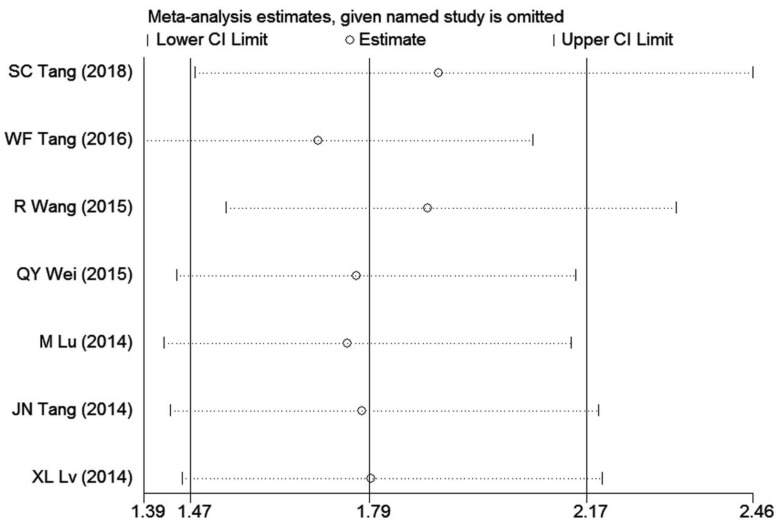
Forests plots of sensitivity analysis for the relationship between high GOLPH3 expression and overall survival (OS) in patients with lung cancer.

**Figure 9:**
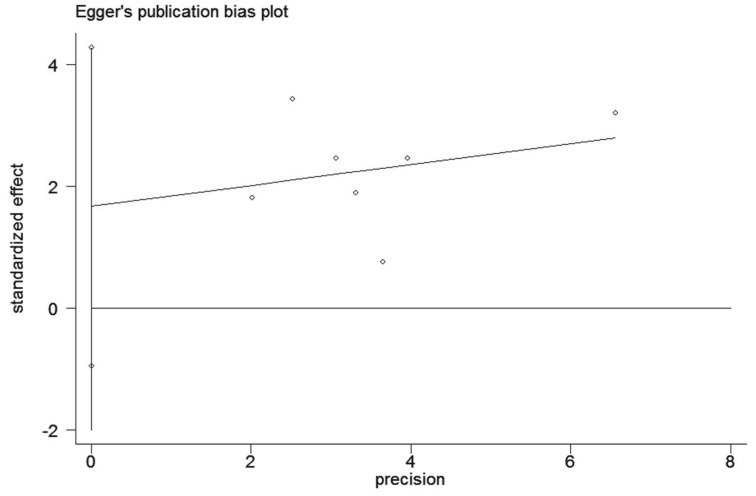
Egger's test for publication bias in GOLPH3 impact on the overall survival (OS) in patients with lung cancer.

In order to examine the stability of the pooled HR in OS analysis, sensitivity analysis was conducted and the results showed that exclusion of individual studies did not modify the overall results (Figure 8). We employed the Egger's test to detect publication bias. The results indicated that no evidence for publication bias was observed in this meta-analysis (Figure 9).

## Discussion

Lung cancer has been a major public health problem and the number of new lung cancer cases is still on the rise largely as a result of an increase in global tobacco use, particularly in the developing countries 1. However, only one third of patients are diagnosed at an early and potentially curable stage, and up to 50% of these patients will develop local or distant recurrences 30, 31. Despite advances in surgical and nonsurgical therapy, the overall survival in cancer patients continues to be poor, with low 5-year survival rates of 21% in women and 15% in men 32. Increasing evidence suggested that the development of tumor biomarkers is crucial for early diagnosis, prognosis assessment and personalized therapy of cancer patients, including the patients with NSCLC. New strategies for identifying sensitive and reliable biomarker for accurate diagnosis and reliable prediction recurrences have attracted a great deal of interest in recent years.

GOLPH3 is a highly conserved protein located in chromosome 5p13, a region that is frequently amplified in multiple solid tumor types 33, 34. It has been demonstrated that GOLPH3 moves between the trans-Golgi network and endosomal structures and serves a vital role in multiple cellular processes, such as trafficking, receptor recycling and protein glycosylation 35, 36. As many proto-oncogenes encode glycosylated proteins involved in tumor cell proliferation, adhesion, invasion, migration, immune recognition and signal transduction 37, GOLPH3 may be associated with tumorigenesis and malignant biological behavior of cancer 35. Further studies showed that GOLPH3 promotes the development of cancer by activating mammalian target of rapamycin (mTOR) signaling, enhancing AKT activity, and decreasing forkhead box O1 (FOXO1) transcriptional activity 13. Recently, it has been reported that GOLPH3 overexpression was involved in cisplatin resistance and the detailed molecular mechanisms were associated with activation of the mitogen-activated protein kinase/extracellular regulated kinase and Wnt/β‑catenin signaling pathways 38. Knockdown GOLPH3 could reduce cell proliferation and induce apoptosis, leading to tumor growth restriction and increased sensitivity of tumor cells to cisplatin 38, 39. Therefore, it is speculated that GOLPH3 plays an essential role in tumor initiation and progression in some types of cancer.

In spite of our efforts to reach an accurate and comprehensive meta-analysis, the meta-analysis also has certain limitations. First, all included studies were retrospective and conducted in Asia with small sample sizes, which may result in potential selection bias. Second, the cut-off values for high/positive expression of GOLPH3 in the eligible studies were different, this inconsistency could have played a role in achieving the outcomes mentioned in this article. Third, several survival data were extracted from the Kaplan-Meier curves according to the method of Tierney et al 19, which could have impact on the credibility of the prognostic effect. Finally, statistical heterogeneity existed among studies. However, random effects model for these analyses and subgroup analysis were conducted to minimize such an impact.

In conclusion, our study demonstrated that GOLPH3 overexpression was positively correlated with adverse clinicopathologic features of NSCLC patients, with a tendency for poor prognosis, suggesting that GOLPH3 plays a vital role in the progression of NSCLC and may serve as a potential prognostic biomarker for NSCLC patients.

## Figures and Tables

**Table 1:** Baseline characteristics of included studies.

Authors	Publication year	Country	Enrollment period	Research design	Resources of samples	Test method	Median follow-up	No. of cases	Male (%)	Clinical stage of patients	score
SC Tang	2018	China	2011-2016	Retrospective	Tumor tissue	Immunohistochemistry	-	60	71.67%	-	7
WF Tang	2016	China	2009-2013	Retrospective	Tumor tissue	Immunohistochemistry	35 months	100	74.00%	stage I- III	6
R Wang	2015	China	2002-2012	Retrospective	Tumor tissue	Immunohistochemistry	-	230	70.87%	stage I- IV	5
QY Wei	2015	China	2001-2005	Retrospective	Tumor tissue	Immunohistochemistry	-	57	70.18%	stage I- IV	6
M Lu	2014	China	2000-2005	Retrospective	Tumor tissue	Immunohistochemistry	52 months	116	58.62%	stage I	6
Y Zhang	2014	China	2001-2007	Retrospective	Tumor tissue	Immunohistochemistry	37 months	145	52.41%	stage I- III	7
JN Tang	2014	China	2005-2012	Retrospective	Tumor tissue	Immunohistochemistry	33.9 months	177	33.90%	stage I- III	8
XL Lv	2014	China	2004-2006	Retrospective	Tumor tissue	Immunohistochemistry	49 months	116	62.07%	-	7

**Table 2:** Prevalence of high expression GOLPH3 staining in cancer tissues and adjacent normal tissues and according clinicopathological characteristics of patients with lung cancer.

Expression of GOLPH3 (positive /all) (N)																				
Author	Adjacent normal tissues	Cancer tissues	Gender		Age (years)			Tumor stage		Tumor differentiation		Lymphatic metastasis		Pathological type		Tumor size				
			Male	Female	< cut-off	≥ cut-off	cut-off value	I-II	III-IV	Well	Moderate to poor	Yes	No	Adenocarcinoma	Squamous cell carcinoma	< cut-off	≥ cut-off	cut-off value	T_1_	T_2_
SC Tang	-	28/60	22/43	6/17	12/32	16/28	60	-	-	-	-	25/43	3/17	14/30	15/30	8/20	20/40	5cm	-	-
WF Tang	0/20	52/100	32/74	20/26	22/39	30/61	60	42/84	10/16	1/18	51/82	20/29	32/71	35/50	17/50	34/61	18/39	5cm	-	-
R Wang	-	230	102/163	42/67	96/145	48/85	65	89/160	55/70	5/12	139/218	54/85	90/145	88/134	54/90	-	-	-	45/86	63/98
QY Wei	1/19	33/57	21/40	12/17	26/41	7/16	65	9/32	24/25	18/32	15/25	28/34	5/23	10/23	23/34	6/11	27/46	3cm	-	-
M Lu	24/57	68/116	42/68	26/48	29/58	39/58	65	-	-	17/31	51/85	-	-	35/66	33/50	-	-	-	24/46	44/70
Y Zhang	33/145	104/145	54/76	50/69	69/94	35/51	65	57/90	47/55	22/40	82/105	59/72	45/73	46/61	48/65	21/31	83/114	3cm	-	-
JN Tang	18/177	116/177	36/60	81/117	56/86	61/91	60	86/122	31/55	-	-	47/61	70/116	45/88	72/89	44/86	73/91	3cm	-	-
XL Lv	12/43	67/116	44/72	23/44	28/52	39/64	65	-	-	19/35	48/81	18/30	49/86	34/66	33/50	-	-	-	30/54	37/62

**Table 3:** Odds ratios of GOLPH3 overexpression in lung cancer tissues according to gender, age and tumor size with subgroup analysis based on study sample size or cut-off.

Variable	Subgroup	OR	95% CI	z-value	p-value	I^2^
gender	overall	1.19	(0.78-1.80)	0.80	0.42	53.6%
	sample size ≥ 100	1.22	(0.77-1.94)	0.84	0.40	59.1%
	sample size < 100	1.05	(0.26-4.26)	0.07	0.94	64.0%
age	cut-off = 60 years	1.13	(0.67-1.91)	0.44	0.66	24.5%
	cut-off = 65 years	0.96	(0.59-1.56)	0.18	0.85	51.8%
tumor size	cut-off = 3 cm	1.98	(0.85-4.60)	1.59	0.11	61.0%
	cut-off = 5 cm	1.48	(0.78-2.83)	1.19	0.24	0.0%
	T descriptor (T_2_ / T_1_)	1.47	(0.99-2.18)	1.93	0.05	0.0%

**Table 4:** Summary of HR with 95%CI for OS and PFS in each study

Author	OS (HR with 95% CI)		PFS (HR with 95% CI)
	Univariate analysis	Multivariate analysis	Multivariate analysis
SC Tang	1.63 (1.21-2.20) / Rep	1.71 (1.25-2.33) / Rep	-
WF Tang	3.91 (1.79-8.50) / Rep	5.67 (1.52-21.11) / Rep	-
R Wang	1.23 (0.72-2.11) / KM	-	-
QY Wei	2.45 (0.93-6.47) / KM	-	-
M Lu	2.23 (1.17-4.20) / KM	1.90 (1.02 -3.53) / Rep	1.83 (1.03 -3.25) / Rep
Y Zhang	-	-	3.58 (1.83-7.01) / Rep
JN Tang	1.86 (1.13-3.04) / Rep	2.19 (1. 29 - 3.73) / Rep	-
XL Lv	1.77 (0.98-3.20) / KM	-	-

OS: Overall survival; PFS: Progression-free survival; HR: Hazard ratios; Rep: Reported in the included studies; KM: Calculated from *Kaplan-Meier* curve.

## Abbreviations

NSCLC: non-small-cell lung carcinoma
GOLPH3: Golgi phosphoprotein 3
HR: hazard ratio
OR: odds ratio
IHC: immunohistochemistry
OS: overall survival
PFS: progression-free survival
mTOR: mammalian target of rapamycin
FOXO1: forkhead box protein O1
